# Case Series of Patients with Opioid Use Disorder and Suicidal Ideation Treated with Buprenorphine

**DOI:** 10.5811/cpcem.2020.10.49610

**Published:** 2020-12-12

**Authors:** Max Spaderna, Melanie Bennett, Rachel Arnold, Eric Weintraub

**Affiliations:** University of Maryland School of Medicine, Department of Psychiatry, Baltimore, Maryland

**Keywords:** Opioid-related disorders, buprenorphine, suicidal ideation, emergency service, hospital

## Abstract

**Introduction:**

Buprenorphine benefits patients with opioid use disorder (OUD) in the emergency department (ED), but its efficacy for OUD patients with suicidal ideation (SI) in the ED is unknown.

**Case Series:**

We present a case series of 14 OUD patients with SI who were given buprenorphine and a referral to outpatient substance use treatment in the ED. All experienced SI resolution, engaged with outpatient services, and remained in outpatient substance use treatment 30 days after ED discharge.

**Conclusion:**

Our data provide evidence for the feasibility of starting buprenorphine in OUD patients with SI in the ED, and suggest that buprenorphine may be useful in helping to resolve SI for these patients. Future research with larger samples is needed.

## INTRODUCTION

The opioid epidemic in the United States has seen an almost sixfold increase in overdose deaths since 1999, a rise that parallels a 30% increase in the suicide rate between 2000–2016.^1^,^2^ While the association between opioid use and suicidal ideation (SI) has been documented, the impact of opioid use on the current escalation in suicides is unclear.^3^ It is likely that suicides caused by opioid overdose are underreported and that many opioid overdose deaths classified as “undetermined” by coroners are suicides.^4^ Treating individuals with opioid use disorders (OUD) must include attention to suicide risk.

The 99.4% increase in opioid-related visits to emergency departments (ED) between 2005–2014 represents an opportunity for EDs to encourage patients already in a vulnerable period to make behavioral changes.^5^,^6^ This includes offering medication-based treatment with methadone and buprenorphine to OUD patients. Studies show that ED-initiated buprenorphine decreases opioid use and increases engagement in outpatient substance use treatment.^7^,^8^ A limitation of these studies is that OUD patients with SI have been excluded, despite some evidence that buprenorphine might reduce SI.^9^

We present observational data on 14 OUD patients with SI who presented to the ED for treatment. Our goals were to explore the feasibility of starting buprenorphine in these patients in the ED, and to determine whether ED-initiated buprenorphine treatment would be associated with improvements in SI and engagement in outpatient substance use treatment.

## CASE SERIES

This study was approved by the medical school’s institutional review board. The 14 patients presented to the ED of a tertiary care hospital between July 2012–August 2018. All met criteria for OUD and reported current SI or a suicide attempt at their index ED visit. All were evaluated by a psychiatrist and offered buprenorphine with a referral to an outpatient substance use treatment program.

We retrospectively reviewed health records of patients referred to the outpatient substance use program affiliated with the medical school. Information collected from the records included the patients’ background characteristics, reasons for ED visit, current substance use, mental health symptoms, buprenorphine use during their ED visit, and engagement in outpatient substance use treatment after ED discharge. Variables collected regarding outpatient substance use treatment included referral to the program during the index ED visit (yes/no); number of days between ED referral to the program and the first appointment (defined as the number of days between discharge from the ED and attendance at the first scheduled appointment); attendance at the first scheduled appointment (yes/no); engagement with the program 30 days after the first scheduled appointment (yes/no, defined as having notes documenting services received on and after day 30); number of medical visits during the 30-day period (defined as visits with a physician to discuss medication and side effects); and number of support visits during the 30-day period (defined as visits with a non-physician to discuss issues besides medication and side effects).

Two of the authors conducted the health record reviews. One (RA) completed the initial review using a data-collection form that matched an Excel (Microsoft Corporation, Redmond, WA) spreadsheet where the data were entered. The second (MB) reviewed the data after these were entered into the spreadsheet and did a comparison with the health record for these variables. Any discrepancy was corrected (if simple), or discussed to reach consensus (if more complex).

Baseline characteristics are summarized in Table 1. Patients were 86% male and 86% Caucasian with a mean age of 41.36 years (standard deviation [SD] =12.18, range 26–60 years). Besides opioids, patients reported current cocaine (n = 10), cannabis (n = 4), and alcohol use (n = 7), along with symptoms of mood (n = 12) and anxiety (n = 6) disorders. Two patients had attempted suicide immediately before presenting to the ED; 12 reported SI as a main reason for their visit.

Buprenorphine use during the ED visit and engagement with outpatient substance use treatment after discharge are summarized in Table 2. The doses of buprenorphine given in the ED ranged from 2–16 milligrams (mg) (mean = 8.00 mg, SD = 3.76). None of the patients required an inpatient hospitalization for SI. All patients received a referral to an outpatient substance use treatment program, attended their first scheduled outpatient substance use treatment visit, and remained engaged with the outpatient substance use treatment program 30 days after their first visit. During the 30 days after their first visit, patients attended between 1–8 medical visits and 7–28 support visits at the outpatient substance use treatment program.

## DISCUSSION

This case series provides evidence for the feasibility and potential benefit of initiating buprenorphine in the ED to OUD patients who present with SI. For all the patients in our observational data, ED-initiated buprenorphine was associated with SI resolution, discharge from the ED, and engagement in outpatient substance use treatment. Given that SI rates remain high among OUD patients in outpatient methadone treatment, medication-based treatment by itself is unlikely to explain why the patients in our series experienced SI resolution.^10^ Although other factors besides buprenorphine initiation might have contributed to SI resolution, our results provide evidence that ED-initiated buprenorphine may be helpful in the treatment of OUD patients who present to the ED with SI.

**CPC-EM Capsule**

What do we already know about this clinical entity?

Buprenorphine initiation in the emergency department (ED) improves engagement in outpatient substance use treatment for opioid use disorder (OUD) patients.

What makes this presentation of disease reportable?

This is the first report of successfully initiating buprenorphine in OUD patients who present to the ED with suicidal ideation (SI).

What is the major learning point?

Buprenorphine initiation and a referral to outpatient substance use treatment may help resolve SI and promote outpatient substance use treatment engagement.

How might this improve emergency medicine practice?

In the ED, buprenorphine initiation and a referral to outpatient substance use treatment may be successful interventions for OUD patients who present with SI.

There could be several explanations for our findings. One explanation might involve buprenorphine’s pharmacology as a partial mu-opioid receptor agonist and kappa-opioid receptor (KOR) antagonist. KOR activation is known to worsen depressive states, and buprenorphine’s antidepressant effect is thought to result from its KOR antagonism, a property methadone lacks.^1112^ Studies have shown that there might be a role for buprenorphine to decrease SI for individuals with and without OUD, and it has been hypothesized that buprenorphine’s anti-suicidal property might result from its KOR antagonism.^913^ Although pharmacologically compelling, this mechanism of action remains speculative, and no evidence links the KOR to SI. Studies are needed to determine whether KOR activation causes or worsens SI.

Another explanation might be that the ED referral to outpatient substance use treatment addressed the non-clinical issue of access to care for OUD treatment that had led these patients to experience SI.^14^ It is notable that all 14 patients attended their first outpatient appointment and remained in treatment for 30 days. Other studies of ED-initiated buprenorphine and referral to treatment have found lower percentages of OUD patients attending their first outpatient appointment.^7^ Many patients did not have to wait long to start outpatient treatment: for the seven patients in our case series whose data are available, the range between the ED referral to outpatient treatment and attendance at the first appointment was 1–5 days. Moreover, by treating both the opioid-withdrawal symptoms and SI, ED-initiated buprenorphine might have illustrated the benefits of continuing buprenorphine after ED discharge to these patients. It might be that the combination of a quick referral to outpatient substance use treatment and the experience of ED-initiated buprenorphine was enough to promote treatment engagement.

Several limitations should be noted. This was a small case series of 14 patients who were not compared with a matched control group that did not receive buprenorphine. We did not examine changes in treatment engagement, opioid use, and SI after 30 days, so we cannot determine how these outcomes might have changed over a longer period of time. We were not able to access data on two variables that might have had an impact on engagement: how patients paid for treatment (eg, insurance, other payment programs, self-pay), or their degree of opioid withdrawal (measured by clinician-rated scores from the Clinical Opiate Withdrawal Scale). It would be useful to know whether any patient experienced self-harm or attempted suicide after the 30 days following their first outpatient substance use visit. Future research should follow patients to examine whether self-harm or suicide attempts occur in the early days of outpatient substance use treatment to evaluate more fully the potential benefit of ED-initiated buprenorphine.

## CONCLUSION

The observational data in our case series provide evidence for the feasibility of starting buprenorphine in OUD patients with suicide ideation in the ED and referring them to outpatient substance use treatment. More rigorous studies are needed to determine the effectiveness of ED-initiated buprenorphine on a larger and more diverse sample of patients.

## Figures and Tables

**Table 1:** Characteristics of 14 patients with opioid use disorder and suicidal ideation at presentation to the emergency department

	Age	Gender	Race	Reasons for ED visit	Self-reported substance use recorded at ED visit	Positive urinalysis at ED visit	Suicidal ideation description recorded at ED visit	Psychiatric symptoms and diagnoses recorded at ED visit
Patient 1	59	Male	Caucasian	suicidal ideation, chest pain	heroin, cocaine	heroin, cocaine	suicidal ideation with plan	depression, bipolar disorder
Patient 2	26	Male	Caucasian	suicidal ideation, alcohol withdrawal	heroin, alcohol	cocaine, benzodiazepines	suicidal ideation with plan	depression
Patient 3	45	Male	Black	suicidal ideation	heroin, cocaine, alcohol, cannabis	heroin, cocaine	unknown	substance-induced mood disorder, depression
Patient 4	35	Male	Caucasian	suicidal ideation, nausea/vomiting, depression	heroin, cocaine, alcohol, cannabis, inhalants	heroin, cocaine, cannabis	suicidal ideation with plan	depression, sleep disturbance, anxiety
Patient 5	60	Male	Caucasian	suicidal ideation, substance use disorder	heroin, cocaine	cannabis	passive suicidal ideation with no plan	depression
Patient 6	27	Female	Caucasian	suicidal ideation with plan	heroin, cocaine, alcohol, benzodiazepines	alcohol	suicidal ideation with plan	post-traumatic stress disorder, substance-induced mood disorder
Patient 7	33	Male	Caucasian	suicide attempt	heroin, cocaine, cannabis, sedatives	cocaine	suicide attempt	depression, sleep disturbance
Patient 8	37	Male	Caucasian	suicidal ideation, groin rash	heroin, cocaine	heroin, cocaine, morphine, fentanyl	suicidal ideation	attention-deficit disorder, anxiety
Patient 9	40	Male	Caucasian	suicidal ideation	heroin	heroin, cannabis, morphine, fentanyl	suicidal ideation	anxiety, depression
Patient 10	28	Male	Caucasian	suicidal ideation, abdominal pain, mild ear pain	heroin, cocaine	cocaine	suicidal ideation with plan	poor sleep, weight loss, irritability
Patient 11	31	Female	Caucasian	suicidal ideation, substance use disorder	heroin, cocaine, cannabis, alcohol	cannabis	passive suicidal ideation	post-traumatic stress disorder, anxiety, bipolar
Patient 12	50	Male	Caucasian	suicidal ideation	heroin, cocaine, alcohol	unknown	passive suicidal ideation	depression
Patient 13	55	Male	Black	suicide attempt	heroin	heroin	suicide attempt	depression, sleep disturbance, appetite disturbance, anxiety
Patient 14	53	Male	Caucasian	suicidal ideation, depression	heroin, alcohol	heroin, cannabis	suicide attempt	loss of appetite, weight loss, low energy

*ED*, emergency department.

**Table 2:** Buprenorphine initiation and outpatient-treatment engagement for 14 patients with opioid use disorder and suicidal ideation at presentation to the emergency department.

	Patient													
	1	2	3	4	5	6	7	8	9	10	11	12	13	14
Buprenorphine prescribed at ED visit	Yes	Yes	Yes	Yes	Yes	Yes	Yes	Yes	Yes	Yes	Yes	Yes	Yes	Yes
Buprenorphine dose in mg* prescribed at ED visit	8mg	4mg	16mg	12mg	12mg	4mg	8mg	8mg	8mg	8mg	8mg	10mg	2mg	4mg
Required inpatient hospitalization for SI	No	No	No	No	No	No	No	No	No	No	No	No	No	No
Self-harm within 30 days after ED visit	None	None	UK	None	None	None	UK	UK	UK	UK	UK	UK	UK	None
Outpatient referral made at ED visit	Yes	Yes	Yes	Yes	Yes	Yes	Yes	Yes	Yes	Yes	Yes	Yes	Yes	Yes
Days between ED referral and 1^st^ appointment	1	1	UK	5	5	4	UK	UK	UK	UK	4	UK	UK	2
Outpatient referral – 1^st^ appointment attended	Yes	Yes	Yes	Yes	Yes	Yes	Yes	Yes	Yes	Yes	Yes	Yes	Yes	Yes
Outpatient referral – engaged 30 days after 1^st^ appointment	Yes	Yes	Yes	Yes	Yes	Yes	Yes	Yes	Yes	Yes	Yes	Yes	Yes	Yes
Outpatient referral – number of medical visits* attended in 30 days	2	2	2	2	2	8	3	2	2	1	3	UK	2	5
Outpatient referral – number of support visits** attended in 30 days	7	19	10	20	23	11	UK	UK	UK	UK	UK	UK	20	28

* Medical visits = visits with a physician, discussion focused on medication and side effects.

** Support visits = visits with a nurse or counselor, discussion focused on issues other than medication and side effects.

*ED,* emergency department; *mg,* milligram; *SI,* suicidal ideation; *UK,* unknown.
